# Developmental-Based Classification of Enkephalin and Somatostatin Containing Neurons of the Chicken Central Extended Amygdala

**DOI:** 10.3389/fphys.2022.904520

**Published:** 2022-05-25

**Authors:** Alessandra Pross, Alek H. Metwalli, Ester Desfilis, Loreta Medina

**Affiliations:** ^1^ Department of Experimental Medicine. University of Lleida, Lleida, Spain; ^2^ Lleida’s Institute for Biomedical Research—Dr. Pifarré Foundation (IRBLleida), Lleida, Spain

**Keywords:** bed nucleus of stria terminalis, central amygdala, intercalated amygdalar cells, stress regulation, embryonic origin, pain regulation

## Abstract

The central extended amygdala, including the lateral bed nucleus of the stria terminalis and the central amygdala, plays a key role in stress response. To understand how the central extended amygdala regulates stress it is essential to dissect this structure at molecular, cellular and circuit levels. In mammals, the central amygdala contains two distinct cell populations that become active (on cells) or inactive (off cells) during the conditioned fear response. These two cell types inhibit each other and project mainly unidirectionally to output cells, thus providing a sophisticated regulation of stress. These two cell types express either protein kinase C-delta/enkephalin or somatostatin, and were suggested to originate in different embryonic domains of the subpallium that respectively express the transcription factors Pax6 or Nkx2.1 during development. The regulation of the stress response by the central extended amygdala is poorly studied in non-mammals. Using an evolutionary developmental neurobiology approach, we previously identified several subdivisions in the central extended amygdala of chicken. These contain Pax6, Islet1 and Nkx2.1 cells that originate in dorsal striatal, ventral striatal or pallidopreoptic embryonic divisions, and also contain neurons expressing enkephalin and somatostatin. To know the origin of these cells, in this study we carried out multiple fluorescent labeling to analyze coexpression of different transcription factors with enkephalin or somatostatin. We found that many enkephalin cells coexpress Pax6 and likely derive from the dorsal striatal division, resembling the off cells of the mouse central amygdala. In contrast, most somatostatin cells coexpress Nkx2.1 and derive from the pallidal division, resembling the on cells. We also found coexpression of enkephalin and somatostatin with other transcription factors. Our results show the existence of multiple cell types in the central extended amygdala of chicken, perhaps including on/off cell systems, and set the basis for studying the role of these cells in stress regulation.

## Introduction

The stress response is triggered by coordinated activation of the neuroendocrine and the autonomic nervous systems, with the participation of specific subsets of neurons of the hypothalamus and brainstem (reviewed by Ulrich-Lai and Herman, 2009). These systems are regulated by the telencephalon, where the central extended amygdala, including the central nucleus of the amygdala and the lateral bed nucleus of the stria terminalis (BSTL), plays critical roles (Phelps and LeDoux, 2005). It appears that the central amygdala is an integration center that plays a key role in systemic stress (involving life threatening challenges), and less so in psychogenic stress that is mostly regulated by the medial amygdala (Ulrich-Lai and Herman, 2009). However, both the central amygdala and the BSTL are very complex in terms of subdivisions, cell composition, chemoarchitecture and connections (Cassell et al., 1986; Gray and Magnuson, 1992; Dong et al., 2001; Dong and Swanson, 2003). Based on its projections, central amygdala regulation of the hypothalamus and brainstem can be direct or indirect by way of the BSTL (Gray and Magnuson, 1987, 1989, 1992; Dong et al., 2001; Davis et al., 2010). By way of its direct projections, the medial part of the central amygdala appears to play a relevant role in phasic (short) fear responses to discrete cues (both conditioned and unconditioned), mostly through control of autonomic and somatic responses (Walker and Davis, 2008; Davis et al., 2010). However, the BSTL (and the central amygdala cells projecting to BSTL) appears to play a prevalent role in sustained (long lasting, anxiety-like) fear responses to contextual conditioned stimuli, mainly by controlling the hypothalamic-pituitary-adrenal axis (Pêgo et al., 2008; Davis et al., 2010). To understand how the central extended amygdala regulates stress it is essential to dissect this structure at molecular, cellular and circuit levels. This has started to be done in mammals, mostly using mouse and rats as models. Tract-tracing studies combined with immunohistochemistry or immunofluorescence showed that both the central amygdala and the BSTL contain different neuropeptidergic cell types involved in internal connections (between central amygdala and BSTL, and viceversa) and/or in projections to the hypothalamus and brainstem. For example, the central amygdala contains enkephalinergic (ENK) neurons and somatostatin (SST) neurons that project to the BSTL (McDonald, 1987; Rao et al., 1987). In addition, the central amygdala and the BSTL contain SST, neurotensin, substance P, and/or corticotropin-releasing factor (CRF) expressing neurons that project to the hypothalamus, the periaqueductal gray, the parabrachial nucleus and the nucleus of the solitary tract (Moga and Gray, 1985; Moga et al., 1989; Gray and Magnuson, 1987, 1992; Gray, 1993). More recently, using optogenetics combined with pharmacological and electrophysiology approaches in mouse, two distinct cell populations of the central amygdala were found to become active (on cells) or inactive (off cells) during conditioned fear responses (Ciocchi et al., 2010). They include two subtypes of inhibitory neurons located in the capsular/lateral subdivisions of the central amygdala, which are able to inhibit each other, and project in a mostly unidirectional manner to output neurons of the medial subdivision of the central amygdala (Ciocchi et al., 2010). It appears that the off cells express the protein kinase C-delta (PKCδ) (Haubensak et al., 2010), many of which are enkephalinergic (more than 40%, Supplementary Table S1 in Haubensak et al., 2010). In contrast, the on cells do not contain PKCδ, but express SST (Penzo et al., 2014). It also appears that in mouse PKCδ positive cells play a role in promoting anxiety-like behavior (Douceau et al., 2022), while SST neurons become active by threat-predicting sensory cues after fear conditioning, and promote passive defensive behaviors (Yu et al., 2016). The ENK/PKCδ cells of the mouse central amygdala have also been involved in stress-induced pain regulation (Paretkar and Dimitrov, 2019). Understanding the neural mechanisms regulating the stress response in non-mammals, and how they help animals to cope with changing environmental conditions, is critical for identifying general principles on stress regulation in vertebrates, but also for improving animal welfare (Broom, 1987). This is a big concern in farm animals, including poultry, which represents one of the most intensive farming systems in the European Union, and produces the second most consumed meat, after pig meat (Augère-Granier and Members' Research Service, 2019). However, brain-behavior relationships are poorly understood in farm animals, including chicken. One of the most challenging problems is to identify in non-mammals the homologues of telencephalic areas known to regulate the hypothalamic-pituitary-adrenal axis and the autonomic nervous system in mammals. This is particularly difficult in birds due to the highly divergent evolution of their telencephalon when compared to that of mammals (Reiner et al., 2004). To solve this problem, our group has been using a very powerful approach based on the evolutionary developmental neurobiology (Medina et al., 2011, 2017). This is based on the fact that, at early embryonic stages, the brain of different vertebrates is more similar, and fundamental divisions-homologous across species-are easily identified based on their topological position together with the combinatorial expression of developmental regulatory genes. This can help to follow these divisions from these early embryonic stages and trace their derived cells throughout development. Since the embryonic origin of cells conditions much of their phenotype, this information is also relevant to better understand the logic behind the connections of different neurons and, overall, the functional networks of the mature amygdala (Medina et al., 2011, 2017; Sokolowski & Corbin, 2012; Morales et al., 2021). We used this approach to identify the cell populations that constitute the central extended amygdala in the chicken and zebra finch telencephalon (Vicario et al., 2014, 2015, 2017). Basically, we found three major cell types derived from the dorsal striatal embryonic division (expressing the transcription factor Pax6), the ventral striatal division (expressing the transcription factor Islet1), and the pallidal division (expressing the transcription factor Nkx2.1), which grouped in different combinations across several areas and nuclei located above the lateral branch of the anterior commissure, between the ventral part of the lateral ventricle and the arcopallium (Vicario et al., 2014, 2015, 2017). From medial to lateral, these included: 1) the BSTL, medially; 2) the peri-intrapeduncular island field (pINP), the oval central nucleus (Ceov), and the perioval zone (Pov), at intermediate levels; and 3) the capsular central amygdala (CeC) and the intercalated cells (ITC), laterally. These cell populations were argued to be homologous to those of the mammalian central extended amygdala (Vicario et al., 2014, 2015, 2017), which include similar cell types with identical embryonic origin (Bupesh et al., 2011). In mouse, SST cells of the telencephalon, including those of the central amygdala, appear to originate from Nkx2.1-expressing progenitors of the pallidal embryonic division, including its ventrocaudal or diagonal subdomain (García-López et al., 2008; Xu et al., 2008; Real et al., 2009; Bupesh et al., 2011; Puelles et al., 2016). Based on this, we suggested that the on cells, previously found to express SST in mouse, have a pallidal origin (Bupesh et al., 2011). Regarding the ENK cells (more than 70% of which express PKCδ in mouse and are thus off cells; Haubensak et al., 2010), based on correlation in their distribution with that of the Pax6 cells, we suggested that they might originate in the dorsal striatal division (Bupesh et al., 2011). Based on the finding of similar cells in birds and turtles, we suggested the presence of on/off cell systems in the central extended amygdala of sauropsids (Medina et al., 2017). However, data on colocalization of ENK or SST with region-specific transcription factors of the subpallium, such as Pax6 (dorsal striatal), Islet1 (ventral striatal) and Nkx2.1 (pallido-preoptic), in cells of the central extended amygdala are missing in mammals and non-mammals. Without this, we cannot know if ENK and SST of the central extended amygdala include one or different subpopulations with different origins. The aim of this study was to analyze coexpression of different transcription factors in ENK or SST cells of the chicken central extended amygdala, and to provide a developmental-based classification of neurons that can be useful in future studies on amygdalar function in chicken.

## Materials and Methods

### Animals

Fertilized eggs of domestic chicks (*Gallus gallus domesticus*; Leghorn strain) were obtained from a commercial hatchery (Granja Santa Isabel, Cordoba, Spain; Authorization ES140210000002), which were incubated at 37.5°C and 55%–60% relative humidity, with rocking. Fertilized eggs were selected by a light test on the day of experiment and only those that contained live embryos were used. The first day of incubation was considered embryonic day 0 (E0).

All animals were treated according to the regulations and laws of the European Union (Directive 2010/63/EU) and the Spanish Government (Royal Decrees 53/2013 and 118/2021) for the care and handling of animals in research. The protocols used were approved by the Committees of Ethics for Animal Experimentation and Biosecurity of the University of Lleida (reference no. CEEA 08-02/19), as well as that of the Catalonian Government (reference no. CEA/9960_MR1/P3/1 for embryos, and CEA/9960_MR1/P4/1 for post-hatchlings).

### Tissue Collection and Fixation

Embryos at embryonic day 16 (E16) and 18 (E18), as well as post-hatchlings until day 2 (P2) were used (*N* = 31). At the right day, animals were anaesthetized as follows. First, for embryos a small hole was made in the egg shell and membrane at the level of the air sac, and then the egg was placed in a camera containing Halothane (2-Bromo-Chloro-1,1,1-trifluoro-ethane, Sigma-Aldrich, Germany; 1 ml Halothane/1,000 ml of chamber volume). Post-hatchlings were also placed in a camera containing Halothane in the same concentration as above, until inducing anesthesia. After, the embryos and post-hatchlings received a euthanasic dose of Dolethal (100 mg/kg of sodium pentobarbital; intraperitoneal). Following this, the animals were perfused transcardially with cold saline solution (0.9% NaCl) containing Heparin (Sigma-Aldrich), followed by phosphate-buffered (PB) 4% paraformaldehyde (PFA 4%, pH 7.4, PB 0.1 M). After dissection and post-fixation (24 h at 4°C), brains were sectioned (100 μm-thick) in coronal plane using a vibratome (Leica VT 1000 S). All brain sections were maintained at 4°C (for short storage) or at−20°C (for longer storage) in hybridization buffer, until being processed as described below.

### Single and Double Chromogenic Labeling: *In Situ* Hybridization and Immunohistochemistry

Some series of brain sections were processed for single *in situ* hybridization, whereas other series of sections were processed for double labeling, doing first *in situ* hybridization, followed by immunohistochemistry.

Brain sections were processed for *in situ* hybridization using digoxigenin-labelled riboprobes, following the procedure previously described by Abellán et al. (2014) and Vicario et al. (2014). The antisense digoxigenin-labelled riboprobes were synthesized using Roche Diagnostics (Switzerland) protocols from cDNAs containing a fragment of the gene of interest:

- Pro-enkephalin (*pENK*; bp 1–862; Genbank accession no. XM_419213.3; BBSRC ChickEST Database, clone ChEST140a9; Boardman et al., 2002).
- Somatostatin precursor (*SST*; bp 1–707; Genbank accession no. NM_205336.1; BBSRC ChickEST Database, clone ChEST114E9; Boardman et al., 2002).

Free floating sections were prehybridized in hybridization buffer (HB; as described by Abellán et al., 2014; Vicario et al., 2014), for 2–4 h at 58°C. Then, the sections were hybridized overnight at 61°C in HB containing 0.5–1 μg/ml of the riboprobe, depending of the age of the embryo. For pENK, the standard HB was used throughout the procedure. For SST, a different, viscous hybridization buffer (HBv) was used during the hybridization step, to optimize the labelling. HBv contained 50% formamide molecular (Sigma-Aldrich), 10% dextran sulfate (Sigma-Aldrich), 1 mg/ml of yeast tRNA (Sigma-Aldrich), 0.2% Tween-20, 2% Denhardt solution (Sigma-Aldrich), and 10% salt solution in RNase and DNase free water (Sigma-Aldrich). The following day, the hybridized sections (both pENK and SST) went through a series of washes: first in HB at 58°C, then in a mix 1:1 of HB and MABT (1.2% Maleic acid, 0.8% NaOH, 0.84% NaCl, and 0.1% Tween-20) at 58°C, and finally in MABT at room temperature. Then, the sections were blocked to avoid unspecific binding, using a blocking solution containing 10% blocking reagent (Roche Diagnostics, Switzerland) and 10% of sheep serum (Sigma-Aldrich) in MABT, for 4 h at room temperature. Following this, the sections were incubated overnight, at 4°C, with a sheep anti-digoxigenin antibody conjugated with alkaline phosphatase (AP coupled anti-DIG, Roche Diagnostics), diluted 1:3,500 in blocking solution, followed by washing with MABT. Signaling was revealed by incubation with nitroblue tetrazolium/5-bromo-4-chloro-3-indolyl phosphate (NBT/BCIP, Roche Diagnostics) for 4–8 h at room temperature. After washing in MABT followed by an overnight step in 4% PFA, some of the sections were then processed for the immunohistochemistry, as follows.

For immunohistochemistry, three different antibodies were used:

- Mouse anti-Pax6, raised against recombinant protein containing aminoacids 1–223 of chick Pax6, made in E. coli (Developmental Studies Hybridoma Bank, University of Iowa, Iowa, IA, United States; catalog reference: Pax6); diluted 1/1,000.
- Mouse anti-Islet1, raised against the C-terminal residues 178–349 of rat Islet1 (Developmental Studies Hybridoma Bank, University of Iowa, Iowa, IA, United States; catalog no. 40.2D6); diluted 1/1,000.
- Rabbit anti- Nkx2.1 (anti- TTF-1), raised against the N-terminal residues 110–122 of rat Nkx2.1 (Biopat Immunotechnologies, Italy; catalog no. PA0100); diluted 1/4,000.

Briefly, sections were first processed to inhibit endogenous peroxidase activity by incubating in 1% H_2_O_2_ and 10% methanol in phosphate buffered saline (PBS) for 30 min. Then, the tissue was permeabilized by washing with PBS containing 0.3% Triton X 100 (PBST; pH 7.4; 0.1 M), followed by an incubation with a blocking solution, containing 10% normal goat serum (NGS; Vector Laboratories Ltd., United Kingdom) and 2% of bovine serum albumin (BSA) in PBST, for 1 h at room temperature. Then, the sections were incubated in the primary antibody, diluted in blocking solution, for about 64 h at 4°C and gentle agitation. After incubation in the primary antibody, the sections were rinsed in PBST and then incubated in a biotinylated secondary antibody diluted in PBST overnight and under gentle agitation.

The secondary antibodies used were:

- Goat anti-mouse, biotinylated, diluted 1/200 (Vector, Burlingame, CA, United States).
- Goat anti-rabbit, biotinylated, diluted 1/200 (Vector, Burlingame, CA, United States).

Then, the sections were rinsed and incubated with the Vectastain Elite ABC Kit (PK- 6100, Vector Laboratories). After rinsing, the immunoreactivity was revealed by a color reaction, incubating the sections in a DAB solution (SIGMAFAST, 3,3′-Diaminobenzidine tablets, Sigma-Aldrich) diluted in water, following the manufacturer instructions. Finally, the sections were rinsed in Tris buffer (0.05 M, pH 8) and then mounted on gelatinized glasses, dehydrated and cover slipped with Permount (Fisher Scientific, United States).

### Double and Triple Fluorescent Labeling: Fluorescent *In Situ* Hybridization and Immunofluorescence

To check if there is coexpression of different markers at cellular level, different series of parallel sections were processed either for double immunofluorescence or for indirect fluorescent *in situ* hybridization combined with single or double immunofluorescence.

The protocol used was previously described by Morales et al. (2021) and Metwalli et al. (2022). Briefly, floating sections were pre-hybridized in HB for 2–4 h at 58°C. Then, the sections were hybridized overnight at 61°C in the HB or HBv buffers previously described containing 1 μg/ml of the riboprobes *pENK* or *SST* (as explained above). The following day, the sections were washed with HB for 30 min at 58°C. After that, a series of washes were done using saline sodium-citrate buffer (SSC; pH 7.5, 0.2 M), 3 times for 20 min, at 58°C, followed by 1 wash in the same buffer for 15 min at room temperature and then 1 wash with Tris buffer (TB, 0.1 M, pH 8) for 15 min at room temperature. The activity of the endogenous peroxidase was inhibited as described above, diluting the hydrogen peroxide in TB. After, the sections were washed with Tris-NaCl-Tween buffer (TNT; 10% TB, pH 8, 0.1 M; 0.9% NaCl; 0.05% Tween-20) for 15 min at room temperature (RT). Following this, the sections were incubated in a blocking solution (TNB) consisting of 20% BBR and 20% of sheep serum in TNT for 2–4 h at RT, followed by incubation in sheep anti-digoxigenin antibody conjugated to the peroxidase enzyme (anti-DIG POD; diluted 1:200; Roche Diagnostics, Basel, Switzerland) in TNB, overnight at 4°C and under gentle agitation. After washing with TNT 3 times for 10 min each, the slices were incubated in Cy3 tyramide complex (AATBioquest, United States), prepared in TB containing 0.003% of H_2_O_2_ (diluted 1/50 and 1/200 for pENK and SST, respectively), for 10 min. Finally, the sections were rinsed in TB and processed for single of double immunofluorescence to detect the same epitopes previously described (Pax6, Islet1 or Nkx2.1).

After tissue permeabilization and blocking of unspecific binding (the same described above for the immunohistochemistry), the sections were incubated in the primary antibodies previously described (rabbit anti-Nkx2.1, diluted 1/4,000; mouse anti-Pax6, diluted 1/100; mouse anti-Islet1, diluted 1/100), and combining them in different ways to obtain the triple labelling staining. Following this, sections were washed and then incubated in one or two of the following fluorescence secondary antibodies, diluted in PBST overnight at 4°C.

- Alexa fluor 488 goat anti-mouse Ig (G+L), Invitrogen-Thermo Fisher Scientific (United States), diluted 1/500
- Alexa fluor 488 goat anti-rabbit Ig (G+L), Invitrogen-Thermo Fisher Scientific (United States), diluted 1/500
- Alexa fluor 405 goat anti-rabbit Ig (G+L) Invitrogen-Thermo Fisher Scientific (United States), diluted 1/500.

Finally, the sections were rinsed in PBS, mounted as explained above, and cover slipped using an antifading mounting medium with or without DAPI (Vectashield Hardset Antifade mounting medium and Vectashield Hardset Antifade mounting medium with DAPI, Vector Laboratories Ltd., United Kingdom).

### Digital Photographs and Figures

Digital microphotographs from chromogenic experiments were taken on a Leica microscope (DMR HC, Leica Microsystems GmbH) equipped with a Zeiss Axiovision Digital Camera (Carl Zeiss, Germany), using ×1.6 and ×5 magnification objectives. Serial images from fluorescent material were taken with a confocal microscope (Olympus FV1000, Olympus Corporation, Japan) using ×10 and ×40 objectives; Z-series stacks were taken at 2 μm-step to allow analysis of co-expression. The fluorescent images were adjusted for brightness and contrast, and extracted using Olympus FV10-ASW 4.2 Viewer (Olympus Corporation). Some of the images at ×40 were used to estimate degree of colocalization in selected areas. Counting was done manually using ImageJ Fiji (Schindelin et al., 2012) at a single z-level, selected for displaying the best labeling for all markers. All the figures were mounted using CorelDraw 2012 (Corel Corporation, Canada).

## Results

### Enkephalinergic Cells

**FIGURE 1:**
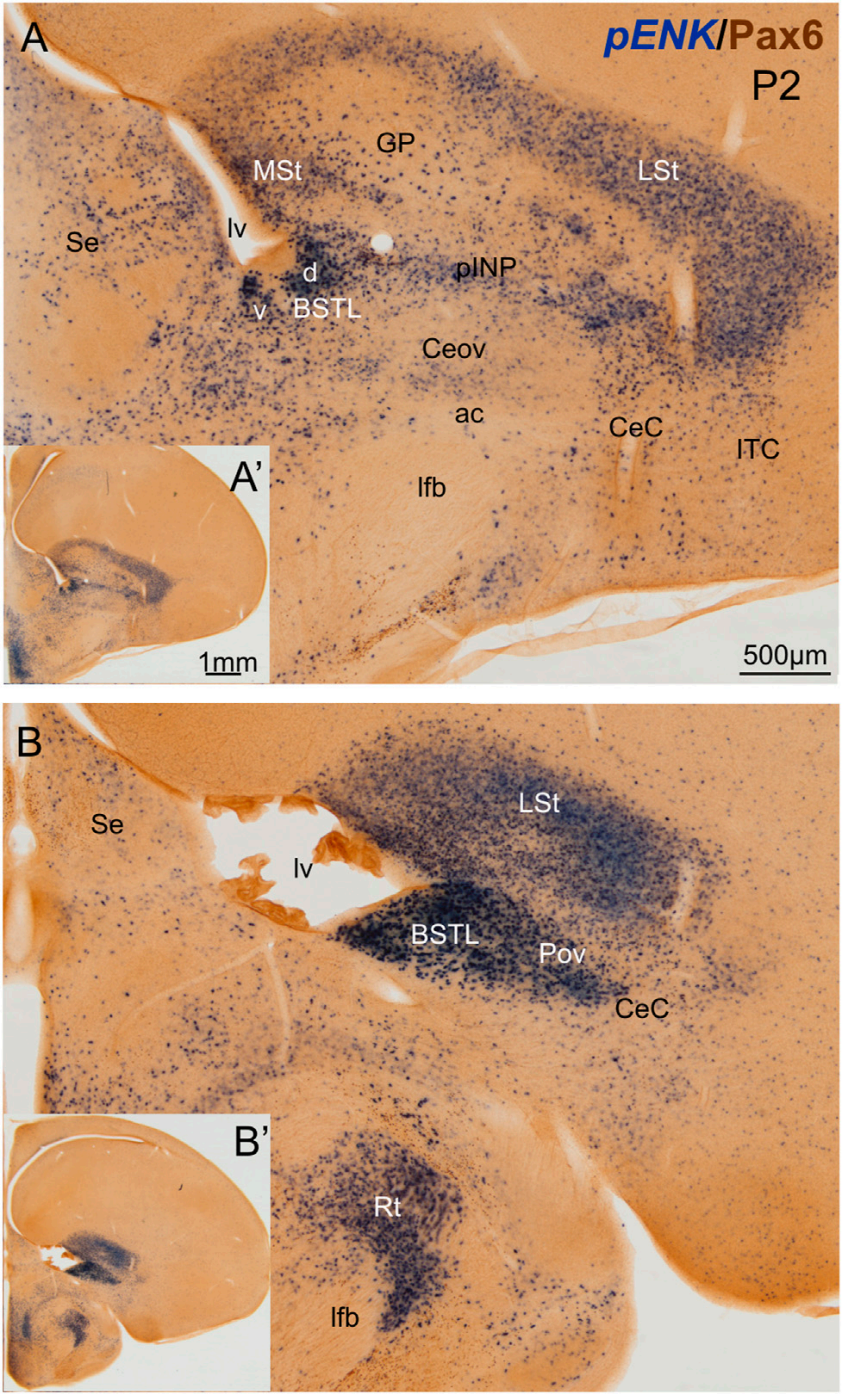
Chromogenic double labeling of pro-enkephalin (*pENK*) and Pax6 in the chicken central extended amygdala at P2. **(A,B)** Details of the subpallium, with the central extended amygdala, taken from frontal sections (insets) of the chicken embryonic telencephalon, at commissural **(A)** and post-commissural **(B)** levels, hybridized for *pENK* (blue color) and immunostained for Pax6 (brown color). Note the high amount of *pENK* cells in the BSTL and pINP. Both areas also contain many Pax6 cells, which are better appreciated in the fluorescent images (Figures 2,5,7). See text for more details. For abbreviations see list. Scale: bar in **(A)** = 500 μm [applies to **(A,B)**]. **(A′)** = 1 mm (applies to **(A′,B′)**].

We first analyzed the distribution of ENK cells in the central extended amygdala of chicken, ranging from E16 to P2. As previously described (Vicario et al., 2014), ENK cells were abundant and densely grouped in the BSTL and pINP, moderately abundant and sparser in CeC and ITC, and scarce in Ceov (Figure 1). We also observed that the BSTL was particularly complex and showed variations along its antero-posterior, mediolateral, and dorsoventral axes. Based on ENK cell distribution, it showed dorsal and ventral subdivisions. The first was larger at anterior levels (Figure 1A), while the second became predominant at posterior levels (Figure 1B). In the dorsal BSTL subdivision, ENK cells were more densely grouped in the intermediate zone of the nucleus, and sparser in the lateral zone (Figure 1A). In the dorsal BSTL subdivision, the periventricular zone was poor in ENK cells, and the ENK cells found in the intermediate zone showed continuity with those in the ventralmost part of the striatal division. In the ventral BSTL subdivision, ENK cells were densely grouped from periventricular to lateral levels. This was especially visible at posterior levels, when pINP had already disappeared (Figure 1B), and ENK cells of BSTL were continuous with those of the perioval zone (Pov) (see also Vicario et al., 2014).

Double chromogenic labeling of ENK with Pax6, Islet1 or Nkx2.1 confirmed overlapping of ENK cells with: 1) Pax6 cells mainly in CeC, pINP and BSTL; 2) Islet1 cells mainly in pINP and BSTL; and 3) Nkx2.1 cells mainly in Pov and BSTL. To know if there is coexpression of ENK and any of the transcription factors, we carried out double and triple fluorescent labeling in animals ranging from E16 until P0 (N = 16; 2 E16, 13 E18, 1 P0).

#### Double Fluorescent Labeling of Enkephalin and Pax6

**FIGURE 2:**
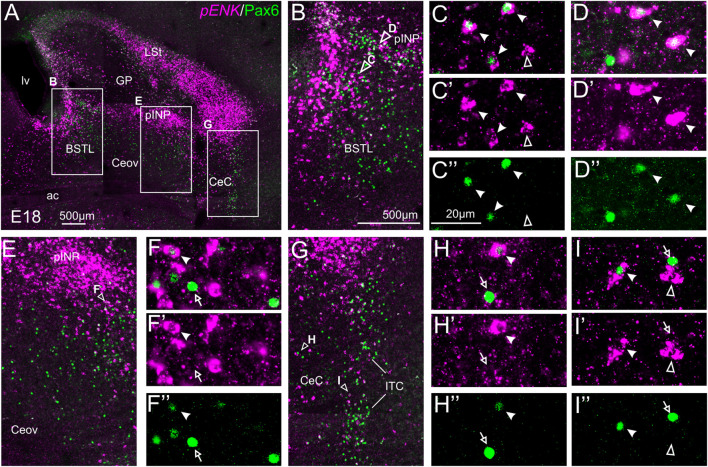
Double fluorescence labeling of pro-ENK (*pENK*) and Pax6 in the chicken central extended amygdala at E18. **(A)** General view (×10 objective) of the subpallium in a frontal section at the level of the anterior commissure, processed for indirect fluorescent *in situ* hybridization for *pENK* (magenta) and immunofluorescence for Pax6 (green). **(B,E,G)** show amplifications of the squared areas in **(A)**, at the level of the BSTL and medial pINP **(B)**, lateral pINP **(E)** or CeC and ITC **(G)**. Details (×40 objective, only one single z level of the confocal stack) of the areas pointed with arrowheads in **(B,E)**, and **(G)** are shown in **(C–C′′)** (for BSTL), **(D–D′′)** (for medial pINP), **(F–F′′)** (for lateral pINP), **(H–H′′)** (for CeC) and **(I–I′′)** (for ITC) (merged plus separate magenta and green channels are shown). In these details, cells coexpressing *pENK* and Pax6 are pointed with a filled arrowhead, cells single labeled for *pENK* are pointed with an empty arrowhead, while cells single labeled for Pax6 are pointed with an empty arrow (only a few examples are pointed). See text for more details. For abbreviations see list. Scale bars: **(A)** = 500 μm; **(B)** = 500 μm [applies to **(B,E,G)**]; **(C′′)** = 20 μm [applies to **(C–C′′,D–D′′,F–F′′, H–H′′,I–I′′)**].

We did indirect *in situ* hybridization for ENK (magenta in Figure 2) combined with immunofluorescence for Pax6 (green in Figure 2). Observation with the 10X objective allowed confirmation of the known labeling patterns of ENK and Pax6 in the subpallium (Figure 2A). Analysis at higher magnification (with 40X objective) allowed distinction of double labeled cells in some of the areas with overlapping of both markers, as follows. Many cases of cells coexpressing ENK and Pax6 were found in the intermediate and lateral zones of dorsal BSTL and medial part of pINP (Figure 2B, details in Figures 2C–C′′,D–D′′). In these areas, about half of the ENK cells coexpressed Pax6. More laterally, the pINP also contained a few cases of double labeled cells (Figure 2E, detail in Figures 2F–F′′). Even more laterally, some cases of double labeled cells were observed in the CeC and ITC (Figure 2G, details in Figures 2H–H′′,I–I′′). Across the areas, some of the double labeled cells showed light intensity of Pax6 immunofluorescence, while other cells were intensely labeled for this transcription factor (for example, Figures 2H–H′′). In all of these areas, we also observed many single labeled ENK cells and Pax6 cells.

#### Double Fluorescent Labeling of Enkephalin and Islet1

**FIGURE 3:**
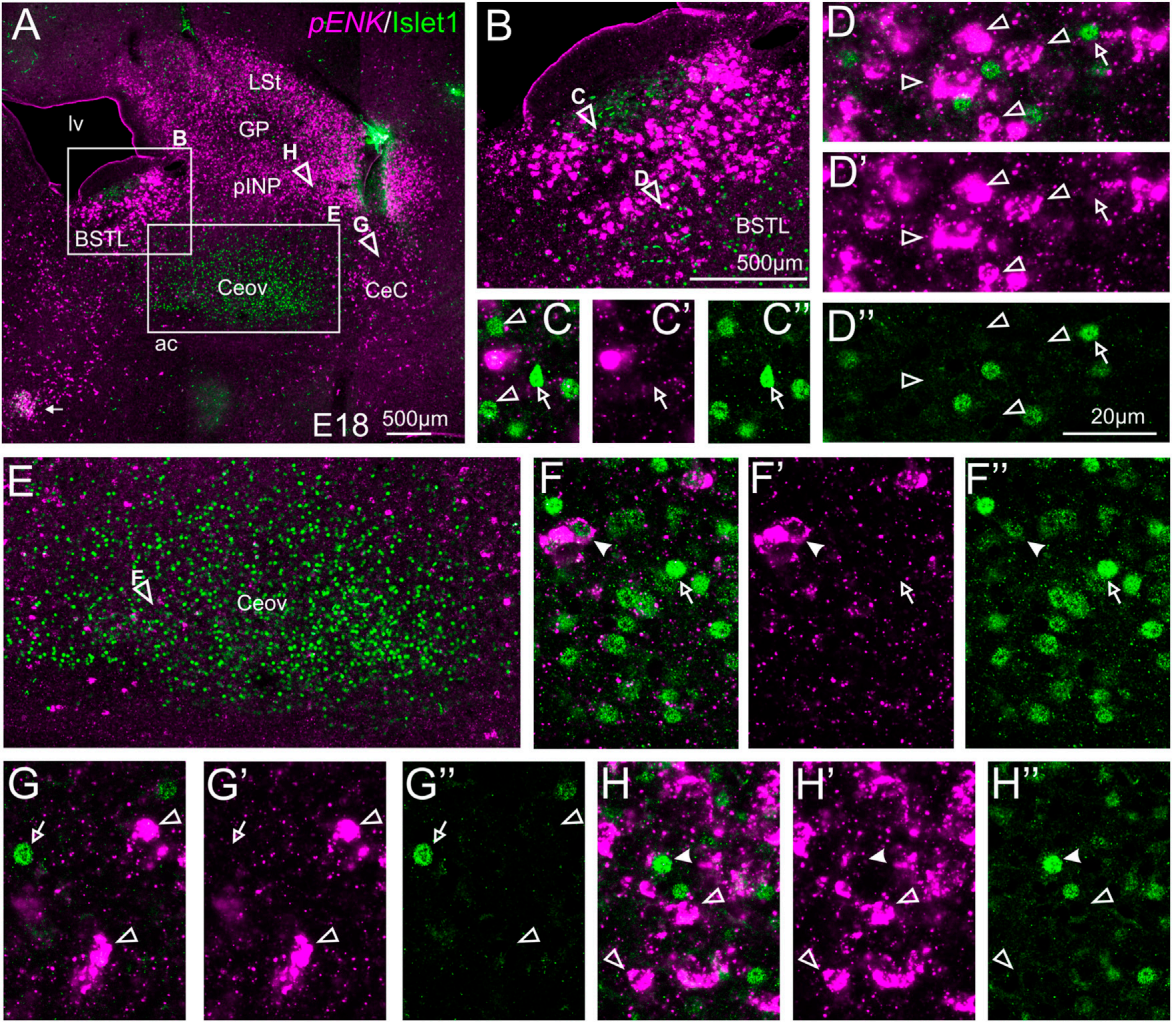
Double fluorescence labeling of pro-ENK (*pENK*) and Islet1 in the chicken central extended amygdala at E18. **(A)** General view (10X objective) of the subpallium in a frontal section at the level of the anterior commissure, processed for indirect fluorescent *in situ* hybridization for *pENK* (magenta) and immunofluorescence for Islet1 (green). **(B,E)** show amplifications of the squared areas in **(A)**, at the level of the BSTL **(B)** and Ceov **(E)**. Details (×40 objective, only one single z level of the confocal stack) of the areas pointed with arrowheads in **(B,E)** are shown in **(C–C′′)** (for medial zone of BSTL), **(D–D′′)** (for intermediate zone of BSTL), **(F–F′′)** (for Ceov). Additional details of the areas pointed with arrowheads in A are shown in **(G–G′′)** (for CeC) and **(H–H′′)** (for pINP) (merged plus separate magenta and green channels are shown). In these details, cells coexpressing *pENK* and Islet1 are pointed with a filled arrowhead, cells single labeled for *pENK* are pointed with an empty arrowhead, while cells single labeled for Islet1 are pointed with an empty arrow (only a few examples are pointed). See text for more details. For abbreviations see list. Scale bars: **(A)** = 500 μm; **(B)** = 500 μm [applies to **(B,E)**]; **(D′′)** = 20 μm [applies to **(C–C′′,D–D′′,F–F′′,G–G′′,H–H′′)**].

We also carried out indirect *in situ* hybridization for ENK (magenta in Figure 3) combined with immunofluorescence for Islet1 (green in Figure 3). Using the 10X objective, we first confirmed that the labeling patterns of ENK and Islet1 in the subpallium were in accordance with previous descriptions (Figure 3A, amplifications of the squared areas are shown in Figures 3B,E). Analysis at higher magnification (with 40X objective) (Figures 3C–C′,D–D′′ in BSTL; Figures 3F–F′′ in Ceov; Figures 3G–G′′ in CeC; and Figures 3H–H′′ in pINP) showed extremely few cases of coexpression of ENK and Islet1 in cells of the chicken central extended amygdala, which were almost restricted to Ceov (Figure 3E, detail in Figures 3F–F′′) and intermediate parts of pINP (Figures 3H–H′′).

#### Double Fluorescent Labeling of Enkephalin and Nkx2.1

**FIGURE 4:**
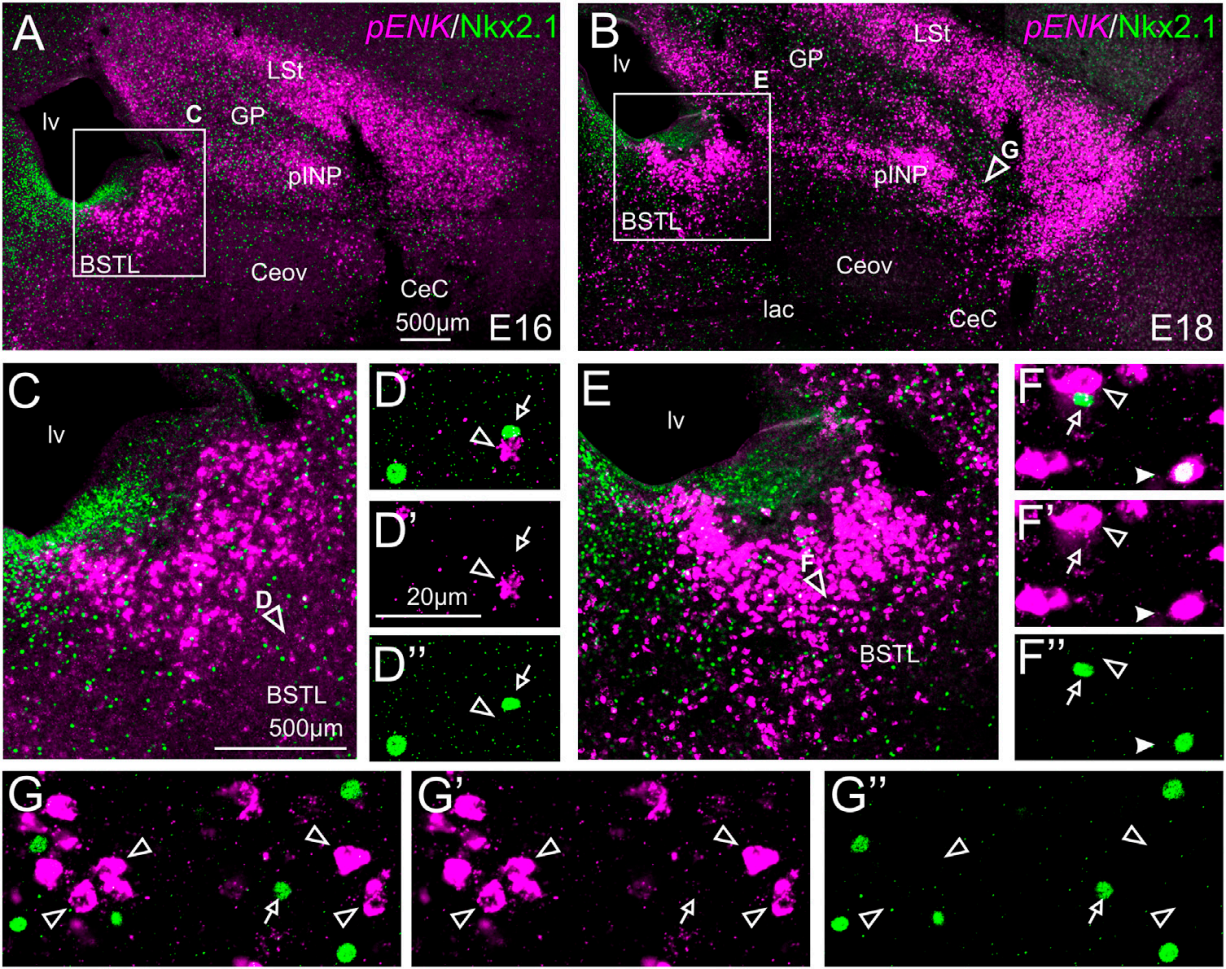
Double fluorescence labeling of pro-ENK (*pENK*) and Nkx2.1 in the chicken central extended amygdala at E16 and E18. **(A,B)** General views (×10 objective) of the subpallium in frontal sections at the level of the anterior commissure, from E16 **(A)** or E18 **(B)** chicken brain, processed for indirect fluorescent *in situ* hybridization for *pENK* (magenta) and immunofluorescence for Nkx2.1 (green). **(C,E)** show amplifications of the squared areas in **(A)** or **(B)**, respectively, at the level of the BSTL. Details (×40 objective, only one single z level of the confocal stack) of the areas pointed with arrowheads in **(C,E)** are shown in **(D–D′′,E–E′′)** (merged plus separate magenta and green channels are shown). Additional details of the areas pointed with arrowheads in **(B)** are shown in **(G–G′′)** (for pINP). In these details, cells coexpressing *pENK* and Nkx2.1 are pointed with a filled arrowhead, cells single labeled for *pENK* are pointed with an empty arrowhead, while cells single labeled for Nkx2.1 are pointed with an empty arrow (only a few examples are pointed). See text for more details. For abbreviations see list. Scale bars: **(A)** = 500 μm [applies to **(A,B)**]; **(C)** = 500 μm [applies to **(C,E)**]; **(D′)** = 20 μm [applies to **(D–D′′, F–F′′,G–G′′)**].

We also performed indirect *in situ* hybridization for ENK (magenta in Figure 4) combined with immunofluorescence for Nkx2.1 (green in Figure 4). Since the downregulation of Nkx2.1 appears to begin in preterm embryos, we also carried out double labeling at an earlier age, E16. Observation with the 10X objective allowed confirmation of the known labeling patterns of ENK and Nkx2.1 in the subpallium (Figure 4A at E16, amplification of the squared area is shown in Figure 4C; and Figure 4B at E18, amplification of the squared area is shown in Figure 4E). Nkx2.1 labeling was more intense at E16 than at E18. Double-labeling of ENK and Nkx2.1 allowed distinction of a “striatal-like” subregion of the dorsal BSTL, not adjacent to the Nkx2.1-expressing ventricular zone (Figures 4C,E). This “striatal-like” subregion was present from rostral to caudal levels of BSTL. The continuity of the ENK cells of the intermediate zone of dorsal BSTL with those found in the striatal division was clearer in this double-labeled material. Analysis at higher magnification (with 40X objective) (Figures 4D–D′′,4F–F′′ in BSTL; and (Figures 4G–G′′ in pINP) showed extremely few cases of cells coexpressing ENK and Nkx2.1, which were restricted to BSTL (filled arrowhead in Figures 4F–F′′), especially at ventral and posterior levels.

#### Triple Fluorescent Labeling of Enkephalin With Pax6 and Nkx2.1

**FIGURE 5:**
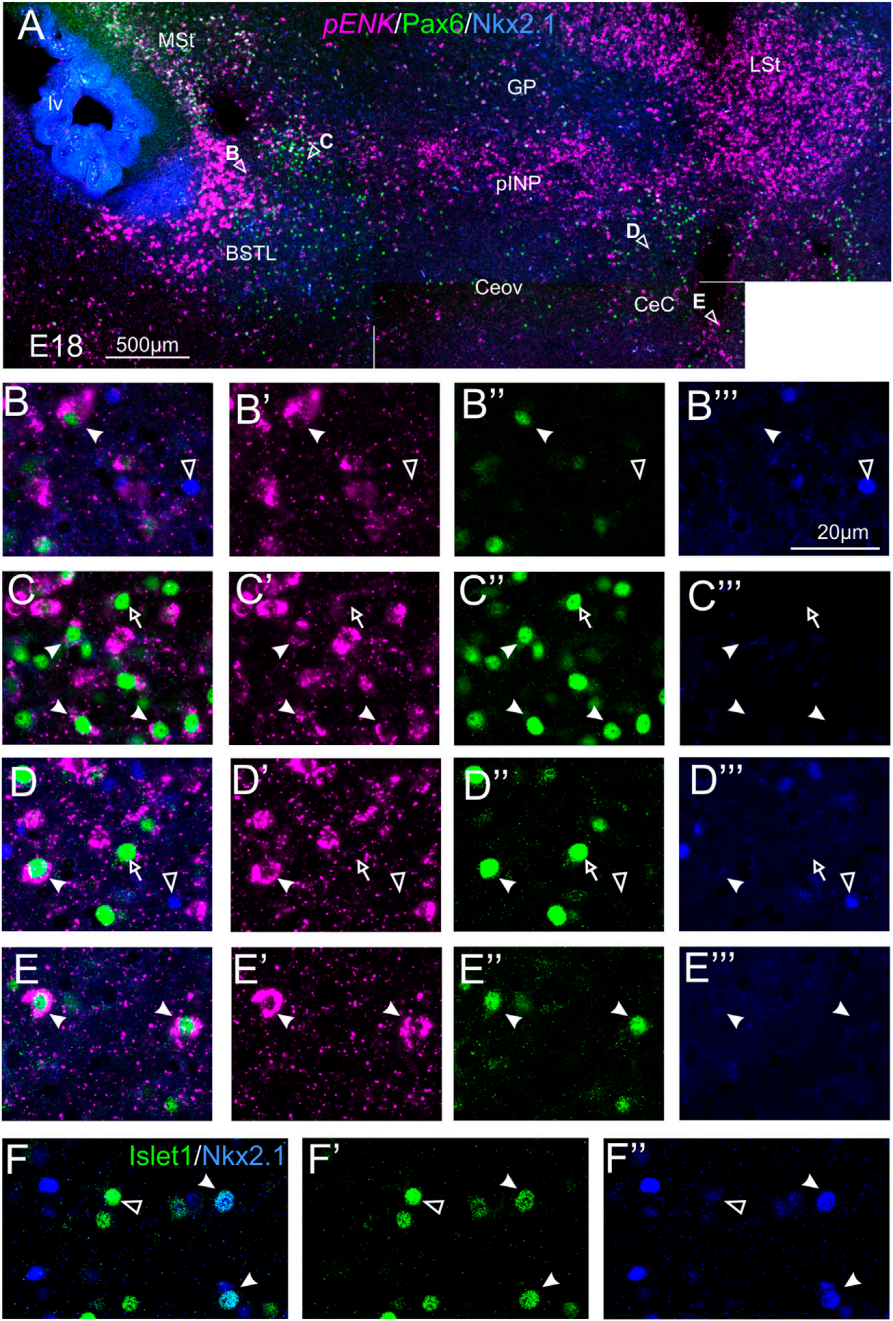
Triple and double fluorescence labeling of pro-ENK (*pENK*), Pax6 and Nkx2.1, or Islet1 and Nkx2.1 in the chicken central extended amygdala at E18. **(A)** General view (×10 objective) of the subpallium in frontal sections at the level of the anterior commissure, processed for indirect fluorescent *in situ* hybridization for *pENK* (magenta), immunofluorescence for Pax6 (green), and immunofluorescence for Nkx2.1 (blue). Details (×40 objective, only one single z level of the confocal stack) of the areas pointed with arrowheads in A are shown in **(B–B‴)** (for BSTL), **(C–C‴)** (for medial pINP), **(D–D‴)** (for lateral pINP, and **(E–E′′)** (for CeC) (merged plus separate magenta and green channels are shown). In these details, cells coexpressing *pENK* and Pax6 are pointed with a filled arrowhead, cells single labeled for Pax6 are pointed with an empty arrow, while cells single labeled for Nkx2.1 are pointed with an empty arrowhead (only a few examples are pointed). No coexpression was seen between Pax6 and Nkx2.1. **(F–F′′)**: Details of the BSTL with double fluorescence of Islet1 (green) and Nkx2.1 (blue). Most cells did not show coexpression (empty arrow), but we found very few examples (filled arrow). See text for more details. For abbreviations see list. Scale bars: **(A)** = 500 μm; **(B‴)** = 20 μm [applies to **(B–B‴)** to **(F–F‴)**].

We also performed triple labeling of ENK (magenta, indirect fluorescent *in situ* hybridization), with Pax6 (green) and Nkx2.1 (blue) immunofluorescence (Figure 5). In the triple labeling experiments, we found no cases of coexpression of Pax6 with Nkx2.1 in the central extended amygdala (details in Figures 5B–B‴ for BSTL; Figures 5C–C‴ for pINP; Figures 5D–D‴ for CeC; and Figures 5E–E‴ for ITC; in these images, the empty arrowheads point to examples of Nkx2.1 labeled cells, while the empty arrows and the filled arrowheads point to examples of Pax6 cells), thus showing that they are separate populations. In addition, the triple labeling helped us to confirm the observations with double labeling on abundant ENK/Pax6 colocalization (filled arrowheads in the previous images), but scarce ENK/Nkx2.1 colocalization in cells of the chicken central extended amygdala.

### Somatostatin Cells

**FIGURE 6:**
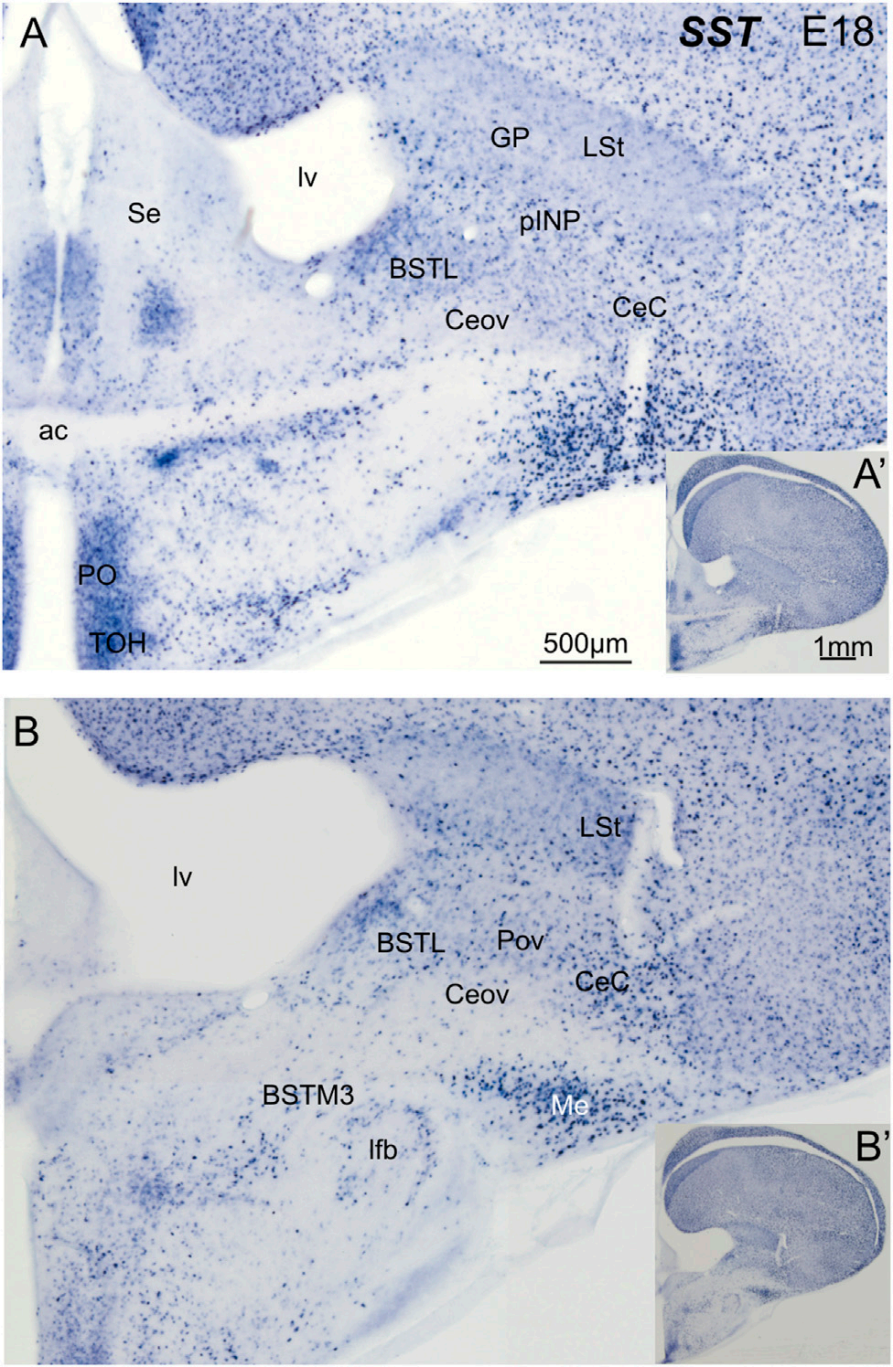
Chromogenic double labeling of somatostatin (*SST*) in the chicken central extended amygdala at E18. **(A,B)** Details of the subpallium, with the central extended amygdala, taken from frontal sections (insets) of the chicken embryonic telencephalon, at commissural **(A)** and post-commissural **(B)** levels, hybridized for *SST* (blue color). Note the presence of SST cells in the BSTL, pINP, Pov and CeC. Many cells are also seen in the medial amygdala, and a subpopulation is also seen in the BSTM3. See text for more details. For abbreviations see list. Scale: bar in **(A)** = 500 μm [applies to **(A,B)**]. **(A′)** = 1 mm (applies to **(A′,B′)**].

We then analyzed the distribution of SST cells in the central extended amygdala of chicken, ranging from E18 until P2 (Figure 6). In contrast to the ENK cells, the SST cells were generally dispersed in the central extended amygdala, and their abundance changed depending on the area. We found SST cells in different areas, including BSTL, pINP, Pov, Ceov, CeC and ITC (Figures 6A,B). SST cells were relatively more abundant in BSTL and CeC, and quite scarce in Ceov (Figures 6A,B). In the BSTL, we observed SST cells in the dorsal and ventral subdivisions. In the dorsal BSTL, SST cells were more abundant in the medial zone, and scattered in the intermediate and lateral zones (Figure 6A). In the ventral subdivision, SST cells were mostly scattered at the medial and lateral zones. To know if there is coexpression of SST and any of the transcription factors, we carried out double fluorescent labeling in animals ranging from E16 until P0 (N = 11; 1 E16, 9 E18, 1 P0).

#### Double Fluorescent Labeling of Somatostatin and Pax6

**FIGURE 7:**
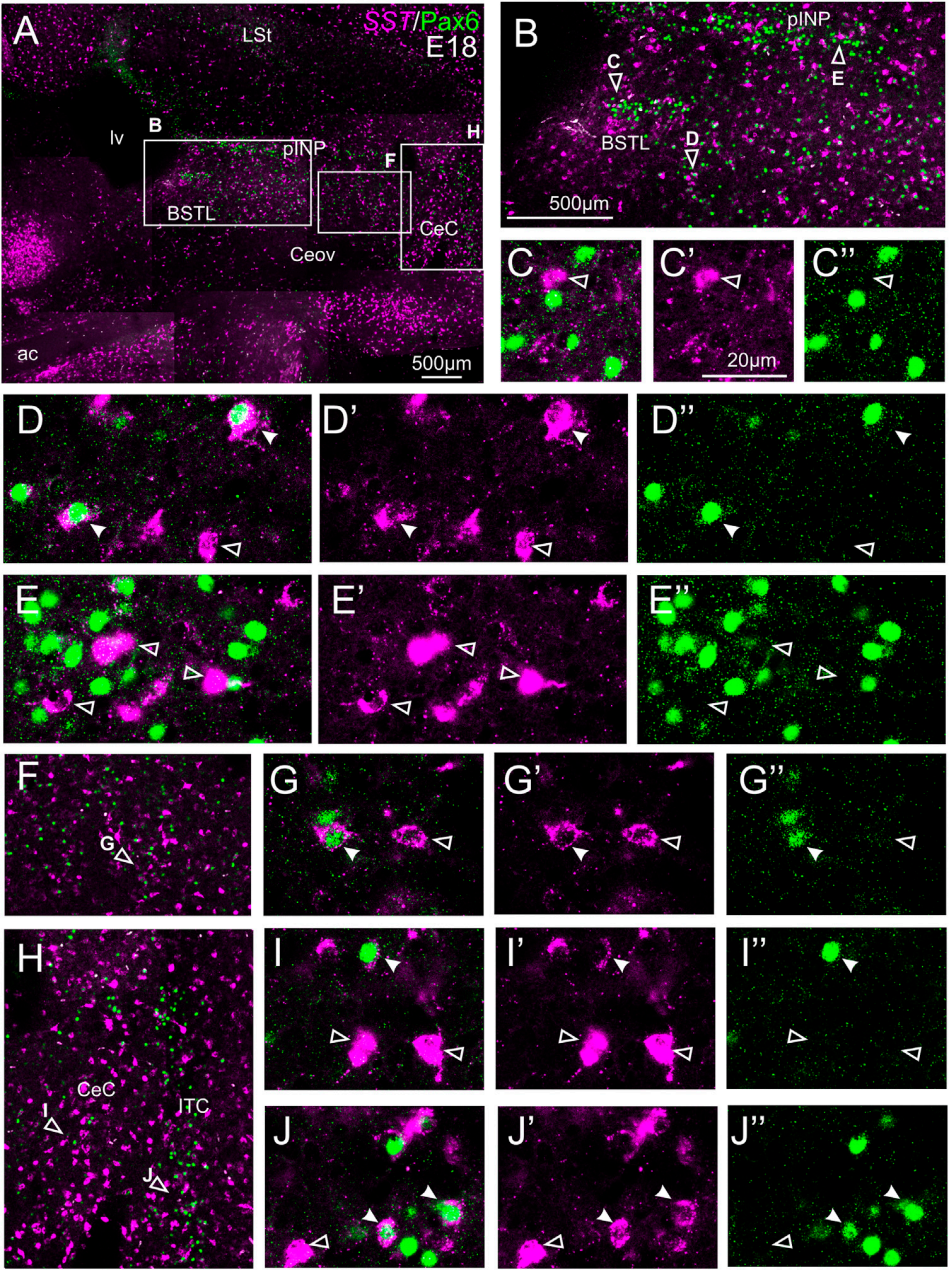
Double fluorescence labeling of somatostatin (*SST*) and Pax6 in the chicken central extended amygdala at E18. **(A)** General view (×10 objective) of the subpallium in a frontal section at the level of the anterior commissure, processed for indirect fluorescent *in situ* hybridization for *SST* (magenta) and immunofluorescence for Pax6 (green). **(B**,**F,H)** show amplifications of the squared areas in **(A)**, at the level of the BSTL and medial pINP **(B)**, Pov **(F)**, and CeC and ITC **(H)**. Details (×40 objective, only one single z level of the confocal stack) of the areas pointed with arrowheads in **(B,F,H)** are shown in **(C–C′′)** (for medial BSTL), **(D–D′′)** (for lateral BSTL), **(E–E′′)** (for medial pINP), **(G–G′′)** (for Pov), **(I–I′′)** (for CeC) and **(J–J′′)** (for ITC) (merged plus separate magenta and green channels are shown). In these details, cells coexpressing *SST* and Pax6 are pointed with a filled arrowhead, and cells single labeled for *SST* are pointed with an empty arrowhead (only a few examples are pointed). See text for more details. For abbreviations see list. Scale bars: **(A)** = 500 μm; **(B)** = 500 μm [applies to **(B,F,H)**]; **(C′)** = 20 μm [applies to **(C–C′′, D–D′′, E–E′′, G–G′′, I–I′′, J–J′′)**].

We did indirect *in situ* hybridization for SST (magenta in Figure 7) combined with immunofluorescence for Pax6 (green in Figure 7). Analysis with the 10X objective allowed confirmation of the known labeling patterns of SST and Pax6 in the subpallium (Figure 7A, amplifications of the squared areas shown in Figures 7B,F,H). Analysis at higher magnification (with 40X objective) allowed distinction of double labeled cells in some of the areas with overlapping of both markers, but not in others. In particular, some double labeled cells were found in the lateral zone of BSTL (Figure 7B, detail in Figures 7D–D′′), in the adjacent Pov (Figures 7B,F, detail in Figures 7G–G′′), in CeC (Figure 7H, detail in Figures 7I–I′′), and ITC (Figure 7H, detail in Figures 7J–J′′). In all of these areas, we also observed many single labeled SST cells and Pax6 cells (empty arrowheads in previous images). In contrast, no double labeled cells were seen in the medial BSTL, pINP and Ceov (Figure 7B, details in Figures 7C–C′′,E–E′′).

#### Double Fluorescent Labeling of Somatostatin and Islet1

**FIGURE 8:**
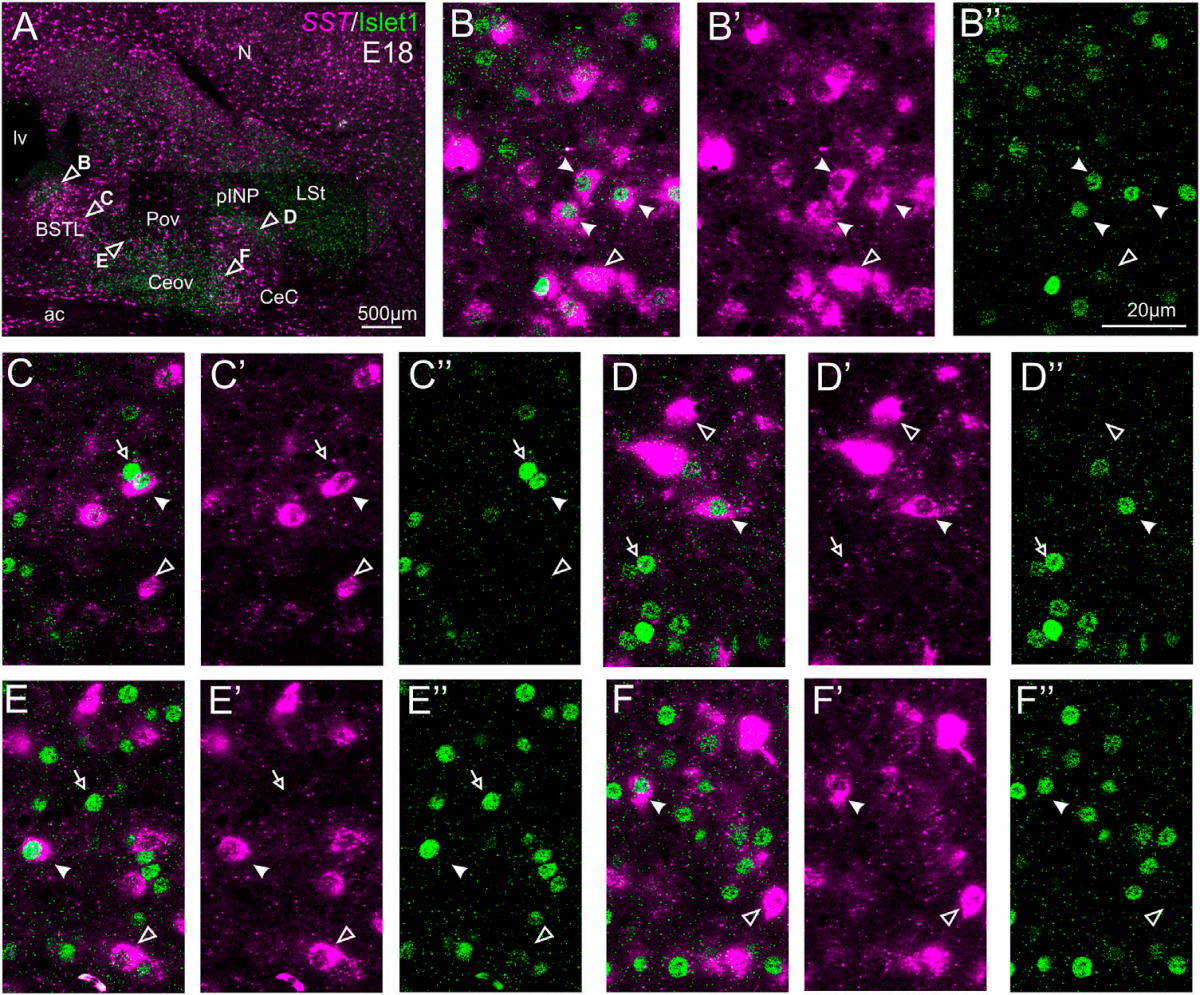
Double fluorescence labeling of somatostatin (*SST*) and Islet1 in the chicken central extended amygdala at E18. **(A)** General view (×10 objective) of the subpallium in a frontal section at the level of the anterior commissure, processed for indirect fluorescent *in situ* hybridization for *SST* (magenta) and immunofluorescence for Islet1 (green). Details (×40 objective, only one single z level of the confocal stack) of the areas pointed with arrowheads in A are shown in **(B–B′′)** (for medial BSTL), **(C–C′′)** (for lateral BSTL), **(D–D′′)** (for lateral pINP), **(E–E′′)** (for medial Pov), and **(F–F′′)** (for lateral Pov) (merged plus separate magenta and green channels are shown). In these details, cells coexpressing *SST* and Islet1 are pointed with a filled arrowhead, cells single labeled for *SST* are pointed with an empty arrowhead, and cells single labeled for Islet1 are pointed with an empty arrowhead (only a few examples are pointed). See text for more details. For abbreviations see list. Scale bars: **(A)** = 500 μm; **(B′′)** = 20 μm [applies to **(B–B′′)** to **(F–F′′)**].

We also carried out indirect *in situ* hybridization for SST (magenta in Figure 8) combined with immunofluorescence for Islet1 (green in Figure 8). Using the ×10 objective, we first confirmed that the labeling patterns of SST and Islet1 agree with previous descriptions (Figure 8A). Analysis at higher magnification (with ×40 objective) allowed distinction of double labeled cells in some of the areas with overlapping of both markers. In particular, we found many cases of double labeled cells in the medial zone of BSTL (detail in Figures 8B–B′′). We also observed a few double labeled cells in the lateral zone of BSTL (detail in Figures 8C–C′′), in pINP (detail in Figures 8D–D′′), and in the medial and lateral parts of Pov (details in Figures 8E–E′′,F–F′′). In all areas, we also observed many single labeled SST cells and Islet1 cells (empty arrowheads and empty arrows, respectively, in previous images).

#### Double Fluorescent Labeling of Somatostatin and Nkx2.1

**FIGURE 9:**
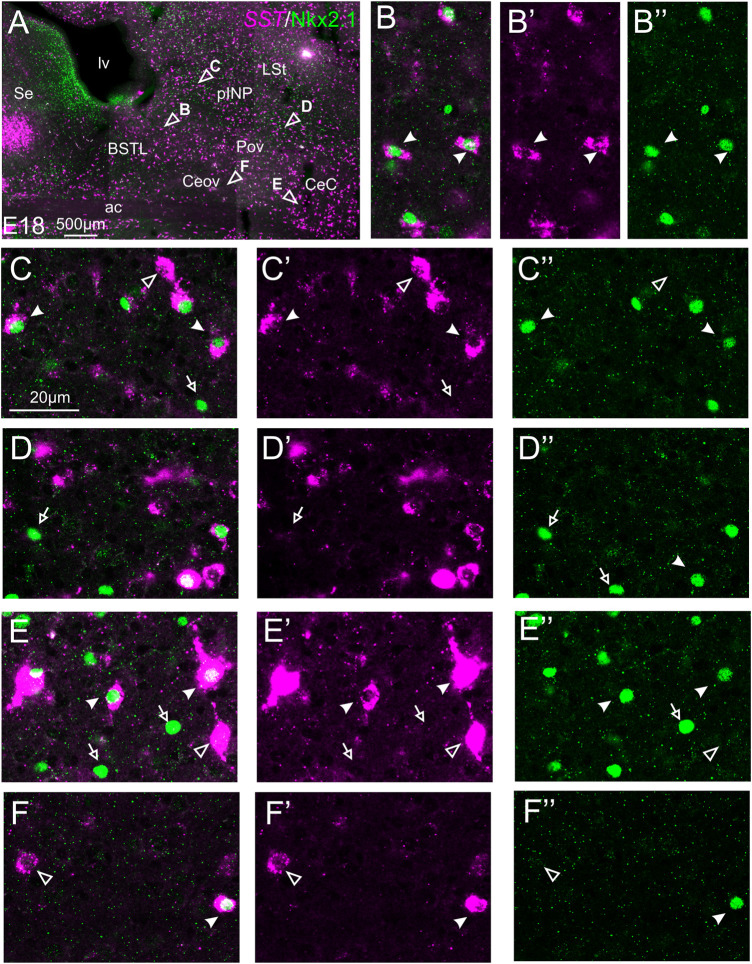
Double fluorescence labeling of somatostatin (*SST*) and Nkx2.1 in the chicken central extended amygdala at E18. **(A)** General view (×10 objective) of the subpallium in a frontal section at the level of the anterior commissure, processed for indirect fluorescent *in situ* hybridization for *SST* (magenta) and immunofluorescence for Nkx2.1 (green). Details (×40 objective, only one single z level of the confocal stack) of the areas pointed with arrowheads in **(A)** are shown in **(B–B′′)** (for lateral BSTL), **(C–C′′)** (for medial pINP), **(D–D′′)** (for lateral pINP), **(E–E′′)** (for CeC), and **(F–F′′)** (for Ceov) (merged plus separate magenta and green channels are shown). In these details, cells coexpressing *SST* and Nkx2.1 are pointed with a filled arrowhead, cells single labeled for *SST* are pointed with an empty arrowhead, and cells single labeled for Nkx2.1 are pointed with an empty arrowhead (only a few examples are pointed). See text for more details. For abbreviations see list. Scale bars: **(A)** = 500 μm; **(C)** = 20 μm [applies to **(B–B′′)** to **(F–F′′)**].

We also performed indirect *in situ* hybridization for SST (magenta in Figure 9) combined with immunofluorescence for Nkx2.1 (green in Figure 9) at E16 and E18. Observation with the 10X objective allowed confirmation of the known labeling patterns of SST and Nkx2.1 in the subpallium (Figure 9A). Analysis at higher magnification (with 40X objective) showed many cases of double labeled cells in the BSTL (detail in Figures 9B–B′′), Pov, pINP (from medial to lateral, details in Figures 9C–C′′,D–D′′, respectively), and CeC (detail in Figures 9E–E′′). For example, in the lateral zone of BSTL, more than half of the SST cells contained Nkx2.1. A few SST/Nkx2.1 double labeled cells were also observed in Ceov (detail in Figures 9F–F′′). In most of these areas, except Ceov, many cases of Nkx2.1 single labeled cells were observed (empty arrows in previous images). Moreover, all of these areas contained a few SST single labeled cells (empty arrowhead in Figures 9C–C′′,E–E′′,F–F′′).

### Colocalization of Transcription Factors

To examine the relationship between cells expressing different transcription factors, we also performed double immunofluorescent labeling of either Pax6 or Islet1 with Nkx2.1. In the double labeling experiments of Pax6 with Nkx.2.1, we found no cases of coexpression, thus confirming the results of triple labeling and showing that Pax6 cells and Nkx2.1 cells are separate populations in the chicken central extended amygdala (see details in Figures 5B–E‴; in these images, the empty arrowheads point to examples of Nkx2.1 cells, while empty arrows and filled arrowheads point to examples of Pax6 cells). Regarding the double labeling of Islet1 with Nkx2.1, most of the cells were single labeled and appear to represent separate populations, but we also observed very few double labeled cells in the lateral part of BSTL (Figures 5F–F′′; filled arrowheads). Based on previous data (Abellán and Medina, 2009; Vicario et al., 2014, 2015), these few Islet1/Nkx2.1 double labeled cells of chicken BSTL likely originate in the preoptic embryonic division.

## Discussion

### A Developmental-Based Classification of ENK and Somatostatin Neurons of the Central Extended Amygdala

Using an evolutionary developmental neurobiology approach, with combinatorial expression of highly conserved region-specific transcription factors and different neuropeptides, we previously identified several areas of the subpallium that constitute the central extended amygdala of chicken and zebra finch, which include the BSTL, the pINP, the Pov, the Ceov, the CeC and the ITC (Vicario et al., 2014, 2015, 2017). These areas contain subpopulations of neurons expressing the transcription factors: Pax6, expressed in cells derived from the dorsal striatal embryonic division; Islet1, expressed in cells derived from the ventral striatal embryonic division; and Nkx2.1, expressed in cells derived from the pallido-preoptic embryonic division (Abellán and Medina, 2009; Vicario et al., 2014, 2015). These areas also contain subpopulations of ENK and SST neurons, which were suggested to have different embryonic origins based on their location in relation to the position of cells expressing different transcription factors, and on comparison to similar data in mouse (Bupesh et al., 2011; Vicario et al., 2014, 2015). For example, ENK cells of the central extended amygdala were suggested to include at least three distinct subpopulations that originate in dorsal striatal (mainly those of CeC and pINP), pallidal or preoptic embryonic domains (mainly those of BSTL), while SST cells were suggested to originate in the pallidal embryonic division (including its ventrocaudal or diagonal domain) (see Figure 10 in Vicario et al., 2015). However, until now there were no data on colocalization of region-specific developmental transcription factors and neuropeptides in cells of the central extended amygdala of any amniote species. In this study, we analyzed coexpression of the transcription factors Pax6, Islet1 or Nkx2.1 in ENK and SST cells of the chicken central extended amygdala, aiming to provide a developmental-based classification of ENK and SST cells that can be used in future studies on amygdalar function in chicken and other amniotes (including mammals).

Regarding the ENK cells, we found coexpression with Pax6 in many cells of BSTL (mainly its lateral zone) and medial pINP, and in a few cells of lateral pINP, CeC and ITC. All of these areas were previously noted to contain abundant Pax6 cells (Vicario et al., 2014). In contrast, we found only very few cases of coexpression of ENK with either Islet1 or Nkx2.1. Our results partially agree with our previous suggestions (Vicario et al., 2015), but also provide new unexpected results. In particular, our results agree with the origin of many ENK cells (those expressing Pax6) of the central extended amygdala in the dorsal striatal embryonic division, but we also found very few cells co-expressing Islet1. Islet1 cells have two possible origins: either the ventral striatal embryonic division or the preoptic area (Vicario et al., 2015). The Ceov and pINP, where very few cases of ENK/Islet1 coexpressing cells were found, are rich in Islet1 cells and we previously found that most of those originate in the ventral striatal embryonic division. However, both also included very few cells expressing Nkx2.1 with apparent pallidopreoptic origin (Vicario et al., 2014, 2015). Since the preoptic embryonic division also produces Islet1 and ENK cells, it is likely that this is the source of the few cells coexpressing both found in Ceov and pINP. In contrast to our previous suggestion that most ENK cells of the BSTL may originate in the pallidal division (Vicario et al., 2014, 2015, 2017), we only found extremely few cases of coexpression of ENK and Nkx2.1 in this and other parts of the central extended amygdala. It is surprising considering the high density of ENK cells in BSTL, especially in its ventrocaudal subdivision. As noted above, a part of these cells co-expresses Pax6 and these likely derive from the striatal division. However, the density of ENK cells in the BSTL is very high, and the ENK/Pax6 cells appear to represent only a small fraction of all ENK cells found in this nuclear complex. It is unclear if our finding of only few cases of coexpression of ENK and Nkx2.1 may be due to the downregulation of Nkx2.1 during intermediate-late embryonic ages (E16-E18 chicken). In agreement with this, we only found very few cases of coexpression of Islet1 and Nkx2.1, which should be expected in cells of preoptic origin (Abellán and Medina, 2009). Additional studies of the central extended amygdala of earlier chicken embryos will be required to further investigate this issue. Alternatively, this could be investigated in transgenic animal models with permanent labeling of Nkx2.1 lineage cells.

With respect to the SST cells, we found that many of them coexpressed Nkx2.1 in most areas of the chicken central extended amygdala, including BSTL, Pov, pINP, CeC and Ceov. This agrees with our previous suggestion that these cells originate in the pallidal embryonic division (Vicario et al., 2014, 2015), and also agrees with previous findings in mouse (Bupesh et al., 2011). However, we previously proposed that most of these cells of the central extended amygdala may originate in the ventrocaudal pallidal (diagonal) division (Vicario et al., 2014, 2015; see also Bupesh et al., 2011; Puelles et al., 2016), but based on the ample distribution of SST cells in dorsal and ventral parts of BSTL, it is possible that other subdomains of the pallidal division also contribute to produce these cells, which would agree with previous findings in mouse regarding the origin of striatal and cortical SST interneurons (Marín et al., 2000; Flames et al., 2007; Fogarty et al., 2007; Xu et al., 2008; Asgarian et al., 2019). Based on data in mouse (Gelman et al., 2011; Asgarian et al., 2019), some SST cells of the chicken telencephalon, including the central extended amygdala, may also originate in the preoptic embryonic domain. These may include the SST cells coexpressing Islet1 found in this study. We also found that not all SST cells of the central extended amygdala of chicken coexpress Nkx2.1. As noted above, this may be due to downregulation of Nkx2.1 at late embryonic stages, but more studies are needed to further investigate if this is so and/or if there is a non-pallidopreoptic source of SST cells for the central extended amygdala. In relation to the latter, in this study we found some cases of SST cells coexpressing Pax6, which origin is unknown. In Nkx2.1-knockout mouse, although most SST cells of the subpallium are missing, a subpopulation of SST cells remains (Marín et al., 2000; Asgarian et al., 2019). It has been suggested that the caudal ganglionic eminence may be the source of such SST cells (Chittajallu et al., 2013), but this finding is controversial (discussed by Asgarian et al., 2019). The mouse caudal ganglionic eminence mostly represents a distinct caudal pole of the striatal embryonic division (i.e., the caudolateral ganglionic eminence) that does not express Nkx2.1 and produces Pax6 cells for the central extended amygdala (Nery et al., 2002; Bupesh et al., 2011). Our results on the existence of SST/Pax6 double labeled cells would agree with the proposal that this caudal pole of the striatal division produces a subpopulation of SST cells for the central extended amygdala, although we cannot discard other sources, such as the prethalamic eminence (PThE), known to give rise to a subpopulation of Pax6 for the extended amygdala in chicken (Abellán and Medina, 2009; see also Alonso et al., 2020, 2021) and mouse (Bupesh et al., 2011; Ruiz-Reig et al., 2017). In addition, other possible sources of SST cells of the central extended amygdala may be the recently described telencephalon-opto-hypothalamic (TOH) domain and the adjacent supraopto-paraventricular hypothalamic (SPV) domain, both of which were found to produce subpopulations of cells expressing the transcription factors Otp and/or Sim1 for the BSTL, the CeC, and ITC in chicken (Metwalli et al., 2022). While SPV and mostly TOH produce subpopulations of cells for the medial extended amygdala in both chicken and mouse, the contribution to the central extended amygdala seems to be specific for chicken, but has not been found in mouse (García-Calero et al., 2021; Morales et al., 2021; Metwalli et al., 2022). It is possible that some of the latter contain SST, since SPV is known to produce SST neurons for the hypothalamus in mouse (Wang and Lufkin, 2000; Díaz et al., 2015), which fail to differentiate in Otp-knockout animals (Wang and Lufkin, 2000). Moreover, our results showed the presence of SST cells in other areas of the extended amygdala that contain cells derived from TOH/SPV, such as the medial bed nucleus of the stria terminalis (BSTM3 subdivision) and the medial amygdala (Figure 6B).

Overall, our results provide a developmental-based classification of the ENK and SST neurons in the chicken central extended amygdala, showing the existence of at least three subtypes of ENK cells and three subtypes of SST cells. According to our data, it seems that a large part of the ENK cells coexpress Pax6 and likely originate in the dorsal striatal embryonic division, while the majority of the SST cells coexpress Nkx2.1 and have a pallidal origin, but additional quantitative studies are needed to investigate the exact proportions. However, these studies would have the limitation of the downregulation in expression of the developmental regulatory transcription factors at late embryonic stages, which combined with the often late expression of adult phenotypic markers would lead to an underestimation of the colocalization. This is likely the case in our study in chicken.

In the mouse central amygdala, most ENK cells coexpress PKCδ (about 70%) and represent a separate cell population from the SST neurons (Haubensak et al., 2010). In fact, ENK/PKCδ and SST occupy partially separate positions in the central amygdala (McCullough et al., 2018). However, while it is true that these cell populations are mostly segregated, there is a very small subpopulation of cells coexpressing PKCδ and SST (McCullough et al., 2018). Thus, we cannot discard the possibility that a small subset of ENK cells coexpress SST in chicken too. These may be the ENK cells found to coexpress Nkx2.1 and/or Islet1 (present results). Nevertheless, in chicken, ENK cells and SST cells show different distribution patterns in the subpallium and likely are mostly segregated. Our data on coexpression with region-specific developmental transcription factors also agree with the major segregation of both types of neuropeptidergic neurons. Regarding the SST cells, in the mouse central amygdala they include several subpopulations, coexpressing tachykinin 2 (substance P), neurotensin and/or corticotropin-releasing factor (McCullough et al., 2018). Based on the embryonic origins of the SST cells (discussed above), these different subpopulations may originate in different subdomains of the pallidal embryonic division, in the preoptic embryonic division, in the PThE, in TOH or in SPV (at least). The existence of different subpopulations of peptidergic neurons with different embryonic origin should be taken into consideration for future studies on the connectivity and function of these different cells. Moreover, cells with origin in the subpallium are GABAergic, while those from PThE, TOH and SPV are glutamatergic (Abellán and Medina, 2009; Ruiz-Reig et al., 2017; Morales et al., 2021). Thus, while most peptidergic neurons of the central extended amygdala originate in the subpallium and are GABAergic, based on the presence of some minor cell subpopulations of the central extended amygdala that may originate in PThE (perhaps some of the SST/Pax6 cells) or the TOH/SPV (perhaps some SST cells that do not express Pax6, Islet1 or Nkx2.1), our results suggest the participation of both GABAergic and glutamatergic networks of the central extended amygdala in the regulation of stress.

### Possible Existence of On/Off Cell Systems for Regulating Stress in the Chicken Central Extended Amygdala

In mammals, the central extended amygdala is known to play a key role in regulation of the stress response (Phelps and LeDoux, 2005; Davis et al., 2010). To understand how the central extended amygdala regulates stress it is essential to dissect this structure at molecular, cellular and circuit levels. Studies in mouse have shown that the central amygdala contains two types of inhibitory neurons that become active (on) or inactive (off) during the conditioned fear response (Ciocchi et al., 2010). These neurons inhibit each other, and project in a mostly unidirectional manner to output neurons of the central amygdala (Ciocchi et al., 2010). It appears that the off cells express the protein kinase C-delta (PKCδ) (Haubensak et al., 2010), and many of them are enkephalinergic (about 40%, Haubensak et al., 2010). In contrast, the on cells do not contain PKCδ, but express SST (Penzo et al., 2014).

In chicken, the BSTL has been shown to become active by stress (Nagarajan et al., 2014), similarly to that of mammals (Davis et al., 2010). Moreover, it projects to the paraventricular hypothalamic nucleus (Atoji et al., 2006), being thus able to regulate the hypothalamic-pituitary-adrenal axis (Smulders, 2021), and receives input from a posterior part of the arcopallium (part of the avian pallial amygdala) (Atoji et al., 2006) that is also involved in control of fear behavior (Saint-Dizier et al., 2009). The subpallial amygdalar area interposed between the arcopallium and the BSTL also projects to the BSTL and seems to belong to the same functional network (Kuenzel et al., 2011). Using an evolutionary developmental neurobiology approach, we recently identified several subdivisions and cell subpopulations within this subpallial amygdala region that, together with the BSTL, appear to form the avian central extended amygdala (Vicario et al., 2014, 2015, 2017). Like that of mammals, this region of chicken also contains a majority of GABAergic neurons (Abellán and Medina, 2009), which originate in identical embryonic subpallial divisions that express Pax6, Islet1 or Nkx2.1 during development (Vicario et al., 2014, 2015). These neurons include subpopulations of ENK and SST neurons. Like in mammals, it is likely that these two types of neurons of the chicken central extended amygdala are involved in inhibitory pathways and inhibit each other to block or release the outputs to the hypothalamus and brainstem centers involved in the stress response. Thus, our data suggest the existence of on/off cell systems in the central extended amygdala of chicken. In addition, our results on coexpression of ENK and SST with different region-specific transcription factors, combined with those from previous experimental studies on the origin of those cells (Bupesh et al., 2011; Vicario et al., 2015), show that many of the ENK cells (including the putative off cells) express Pax6 and originate in the dorsal striatal embryonic division, while most SST cells (including the putative on cells) express Nkx2.1 and derive from the pallidal embryonic division. Based on the presence of Pax6, Islet1 and Nkx2.1 expressing cells in the central extended amygdala of turtles, derived from dorsal striatal, ventral striatal or pallidal embryonic divisions (Moreno et al., 2010), it appears that on/off cell systems may be a common feature in the central extended amygdala of amniotes (discussed by Medina et al., 2017). In the central extended amygdala of amphibians and lungfishes, Pax6 cells of dorsal striatal origin appear to be missing (Moreno et al., 2008; Moreno et al., 2018), suggesting that the on/off cell systems may be an innovation of amniotes, likely contributing to a more sophisticated and plastic regulation of the stress response (Medina et al., 2017).

### Enkephalin Cells of the Central Amygdala and Regulation of Pain

Painful stimuli are known to recruit the amygdala, leading to anxiety-like behavior and to changes in pain sensitivity (Jiang et al., 2014; Thompson and Neugebauer, 2017). Studies in mammals have shown that regulation of pain by the central amygdala is complex, and can lead to either analgesia or algesia depending on the specific cells and subcircuits involved (Veinante et al., 2013; Andreoli et al., 2017; Paretkar and Dimitrov, 2019). Stress can also regulate pain sensitivity, and depending on the duration and intensity can lead to stress-induced analgesia or to hyperalgesia (Parikh et al., 2011; Baker et al., 2019). Stress-induced analgesia can be mediated by endogenous opioids, such as ENK, or by non-opioids (Baker et al., 2019). It appears that more severe, short and continuous stressors involve non-opioid mediated analgesia. On the contrary, less severe, intermittent or long-duration stressors involve opioid-mediated analgesia (Parikh et al., 2011; Baker et al., 2019). The latter type involves activation of opioid receptors in the central amygdala (Zhang et al., 2013). This type of analgesia can also be produced by the chemogenetic activation of ENK neurons of the central amygdala (including the ENK/PKCδ neurons), which results in anxiolysis and increase of pain threshold (Paretkar and Dimitrov, 2019). Activation of these neurons leads to inhibition of output central amygdala neurons that project to the periaqueductal gray (Haubensak et al., 2010; Paretkar and Dimitrov, 2019), a midbrain region critical for orchestrating behavioral responses to internal and external stressors and for modulating pain sensitivity (Oliveira and Prado, 2001; Benarroch, 2012). As a consequence, the ventrolateral periaqueductal gray is disinhibited and becomes active (Paretkar and Dimitrov, 2019). This indirectly acts on the gate control system of pain in the dorsal horn, in the spinal cord, leading to inhibition of ascending nociceptive transmission, which results in analgesia (Baker et al., 2019).

Data on the central neural regulation of pain in birds is limited, but the avian brain and spinal cord contain areas, networks, and neurotransmitter/neuropeptide systems similar to those involved in pain regulation in mammals (Kuenzel, 2007; Baker et al., 2019). It also appears that in young chickens there is a reduction in pain response following exposure to a stressor (Sufka and Hughes, 1991; Feltenstein et al., 2002), which suggests the implication of the amygdala. Our finding of ENK cells in the central extended amygdala of chicken, comparable to those of mammals, open new venues for investigating if in birds these cells also play a role in stress-induced regulation of pain.

## Data Availability

The raw data supporting the conclusions of this article will be made available by the authors, without undue reservation.

## Glossary

ac: anterior commissure
BST: bed nucleus of the stria terminalis
BSTL: lateral division of the BST
BSTM: medial division of the BST
BSTM3: part 3 of BSTM
Ce: central amygdala
CeC: capsular division of the Ce
Ceov: oval nucleus of the Ce
CRF: corticotropin releasing factor
d: BSTLd
EAce: central extended amygdala
ENK: enkephalin
GP: globus pallidus
ITC: intercalated amygdalar cells
lac: lateral branch of the anterior commissure
lfb: lateral forebrain bundle
LSt: lateral striatum
lv: lateral ventricle
Me: medial amygdala
MSt: medial striatum
N: nidopallium
PKCδ: protein kinase C-delta
pINP: peri-intrapeduncular island field
PO: preoptic area
Pov: perioval zone (part of the EAce)
PThE: prethalamic eminence
Rt: reticular prethalamic nucleus
Se: septum
SST: somatostatin
SPV: supraopto-paraventricular hypothalamic domain
TOH: telencephalo-opto-hypothalamic domain
v: BSTLv
